# The Antihyperlipidemic Mechanism of High Sulfate Content Ulvan in Rats

**DOI:** 10.3390/md13063407

**Published:** 2015-05-29

**Authors:** Huimin Qi, Jiwen Sheng

**Affiliations:** Weifang Medical University, No.7166 Baotong Road, Weifang 261053, China; E-Mail: shengjiwen@wfmc.edu.cn

**Keywords:** *Ulva pertusa*, sulfated polysaccharide, antihyperlipidemic, mechanism

## Abstract

Numerous studies have suggested that hyperlipidemia is closely linked to cardiovascular disease. The aim of this study was to investigate the possible antihyperlipidemia mechanism of HU (high sulfate content of ulvan) in high-cholesterol fed rats. Wistar rats were made hyperlipidemic by feeding with a high-cholesterol diet. HU was administered to these hyperlipidemia rats for 30 days. Lipid levels and the mRNA expressions of FXR, LXR and PPARγ in liver were measured after 30 days of treatment. In the HU-treated groups, the middle dosage group of male rats (total cholesterol (TC): *p* < 0.01) and the low-dosage group of female rats (TC, LDL-C: *p* < 0.01) showed stronger activity with respect to antihyperlipidemia. Moreover, some HU groups could upregulate the mRNA expression of FXR and PPARγ and downregulate the expression of LXR. For the male rats, compared with the hyperlipidemia group, the middle dosage HU had the most pronounced effect on increasing the mRNA levels of FXR (*p* < 0.01); low- and high-dosage HU showed a significant inhibition of the mRNA levels of LXR (*p* < 0.01). All HU female groups could upregulate the mRNA expression of PPARγ in a concentration-dependent manner. In summary, HU could improve lipid profiles through upregulation of FXR and PPARγ and downregulation of LXR.

## 1. Introduction

Hyperlipidemia, one of the metabolic diseases, is characterized by an abnormal elevation of lipids and lipoproteins, such as serum triglyceride (TG), total cholesterol (TC) and low-density lipoproteins (LDL) [1,2]. For the treatment of hyperlipidemic patients, niacin is the most frequently prescribed, either alone or with lipid-lowering drugs, such as lovastatin (statin) and gemfibrozil (fibrates). However, recent reports of undesirable side effects (myopathy) of some “super statins” indicate that the scope of improving the potency of this class of drugs may be modest [3]. Thus, it is essential to develop and utilize effective and natural agents that may be beneficial in correcting lipid metabolism and preventing cardiac diseases.

Some natural products, such as essential oil of *Pinus koraiensis* [4], *Citrullus colocynthis* seeds [5] and *Costus speciosus* root [6], were reported to have antihyperlipidemic activity. Especially, many marine resources have attracted attention in the search for antihyperlipidemic compounds to develop new drugs and heath foods. According to reports, chitosan, chitosan derivatives, ulvan and high sulfate content ulvan all showed antihyperlipidemic activity [7,8,9].

**Figure 1:**
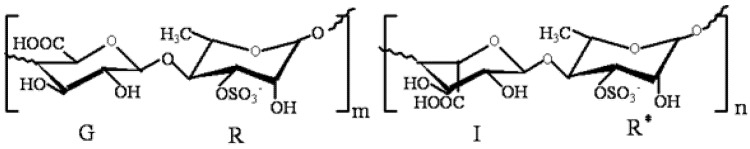
The structure of ulvan, the main disaccharide units [β-d-Glcp A-(1→4)-α-l-Rhap 3s] and [α-l-Idop A-(1→4)-α-l-Rhap 3s]; G: (1→4)-linked β-d-glucuronic acid; R: (1→4)-linked-α-l-rhamnose-3-sulfate (linked with β-d-glucuronic acid); I: (1→4)-linked α-l-iduronic acid; R*: (1→4)-linked-α-l-rhamnose-3-sulfate (linked with α-l-iduronic acid).

The green alga, *Ulva pertusa*, is nutritious, low calorie and abundant in vitamins, trace elements and dietary fibers [10]. Moreover, it is an important marine drug, prescribed in the Chinese Marine Materia Medica [11]. Ulvan, the sulfated polysaccharide comprising the hot-water soluble portion of the cell wall, is one of the main components of *U. pertusa*, and the main disaccharide units are [β-d-GlcpA-(1→4)-α-l-Rhap3s] and [α-l-Idop A-(1→4)-α-l-Rhap 3s]. The main structure of ulvan is shown in Figure 1 [12]. During the last few years, ulvan has been reported to have antihyperlipidemic, antioxidant, antitumor and antiviral activities [12,13,14,15]. The derivative of high sulfate content ulvan (HU) was prepared and showed higher antihyperlipidemic activity than ulvan [9]. However, there is no information on the antihyperlipidemic mechanism of HU so far. Thus, the present investigation aimed to determine the expression of lipid metabolism genes, including FXR, LXR and PPARγ, in rat’s livers, then tried to prove the antihyperlipidemic mechanism of HU.

## 2. Results and Discussion

### 2.1. Results

#### 2.1.1. Body Weight and Food Intakes

**Figure 2:**
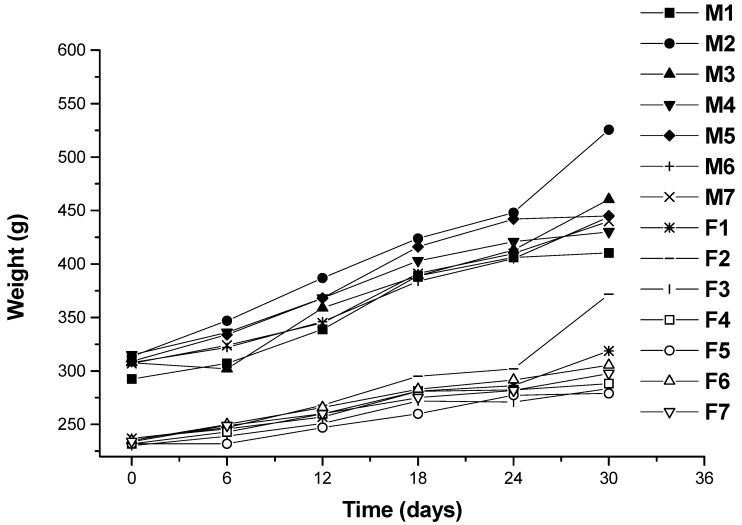
Growth curves for Wistar rats for 30 days. M1: male rats in Group 1 (normal control); M2: male rats in Group 2 (hyperlipidemia); M3: male rats in Group 3 (ulvan, 250 mg/kg); M4: male rats in Group 4 (high sulfate content ulvan (HU), 125 mg/kg); M5: male rats in Group 5 (HU, 250 mg/kg); M6: male rats in Group 6 (HU, 500 mg/kg); M7: male rats in Group 7 (cholestyramine, positive control, 500 mg/kg); F1: female rats in Group 1 (normal control); F2: female rats in Group 2 (hyperlipidemia); F3: female rats in Group 3 (ulvan, 250 mg/kg); F4: female rats in Group 4 (HU, 125 mg/kg); F5: female rats in Group 5 (HU, 250 mg/kg); F6: female rats in Group 6 (HU, 500 mg/kg); F7: male rats in Group 7 (cholestyramine, positive control, 500 mg/kg).

**Figure 3:**
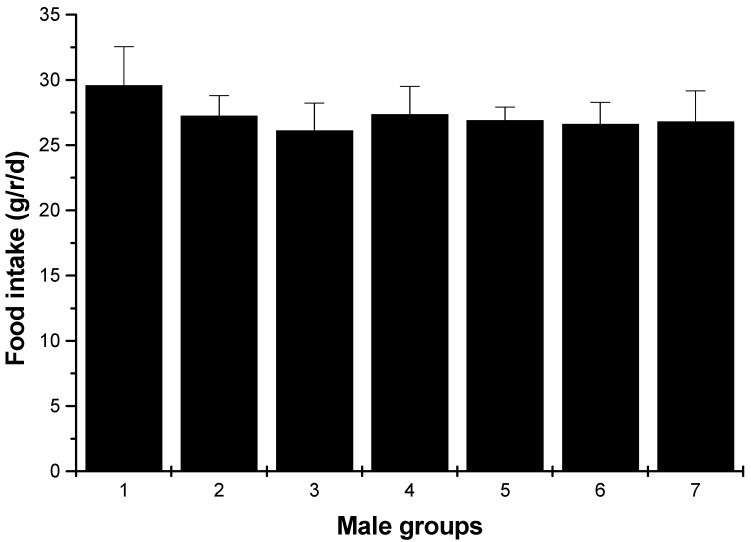
Food intakes of male rats (average, g/rat (r)/day). 1: normal control group; 2: hyperlipidemia group; 3: ulvan group (250 mg/kg); 4: HU low-dose group (125 mg/kg); 5: HU middle dose group (250 mg/kg); 6: HU high-dose group (500 mg/kg); 7: positive group (cholestyramine, 500 mg/kg).

**Figure 4:**
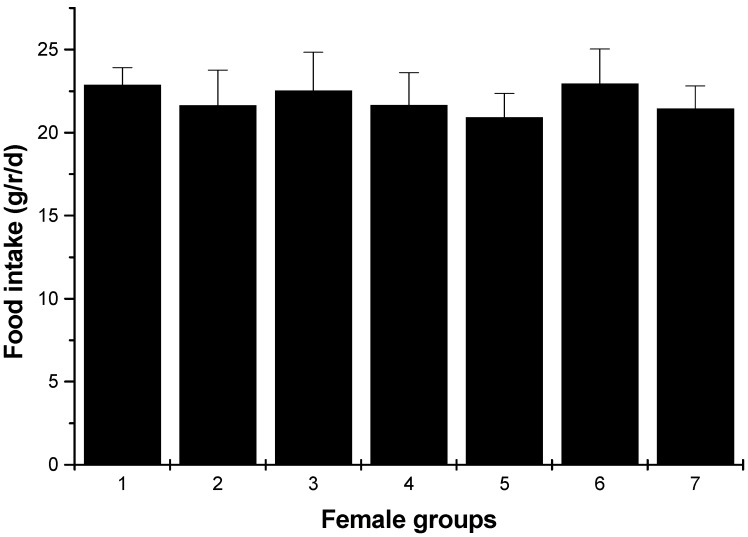
Food intakes of female rats (average, g/rat/day). 1: normal control group; 2: hyperlipidemia group; 3: ulvan group (250 mg/kg); 4: HU low dose group (125 mg/kg); 5: HU middle dose group (250 mg/kg); 6: HU high dose group (500 mg/kg); 7: positive group (cholestyramine, 500 mg/kg).

Changes in body weight during the administration period are shown in Figure 2. Values for all dose groups in both sexes were comparable to those of the controls groups. Except for hyperlipidemia groups, the other groups had no significant differences recorded throughout the administration period. At the end of the experimental period (30 days), both the male and female hyperlipidemia groups showed higher weight than the control group (*p* < 0.05). During the administration period, the averages of food intakes for each group were obtained. The results showed that there was no significant difference in food intakes between the ulvan (U), HU groups and normal control rats (Figure 3 and Figure 4).

#### 2.1.2. Lipid Content in Serum

As shown in Table 1, after 30 days of cholesterol-rich feeding, the serum levels of TC, TG and LDL-C in the hyperlipidemia group were significantly higher than those in the normal control group (*p* < 0.05), whereas the serum levels of HDL-C in the hyperlipidemia group were significantly lower, which indicated that the model was successful inducing hyperlipidemia in rats. For the male rats, compared with the hyperlipidemia group, the results indicated that middle dose HU group (250 mg/kg) had optimal effect on TC (*p* < 0.01), but a lesser impact on LDL-C and HDL-C. However, for the female rats, compared with the hyperlipidemia group, the results proved that the low-dose HU group (125 mg/kg) had an obvious effect with respect to TC and LDL-C (*p* < 0.01). For both male and female rats, the results indicated that the antihyperlipidemic activity was not concentration dependent for HU-fed rats. On the other hand, for the female rats, compared with the control group, the low-dose and middle dose groups had a greater effect with respect to decreasing the TC concentration. On increasing the HDL-C levels, the positive cholestyramine showed stronger activity than U and HU.

#### 2.1.3. FXR mRNA Expression

**Figure 5:**
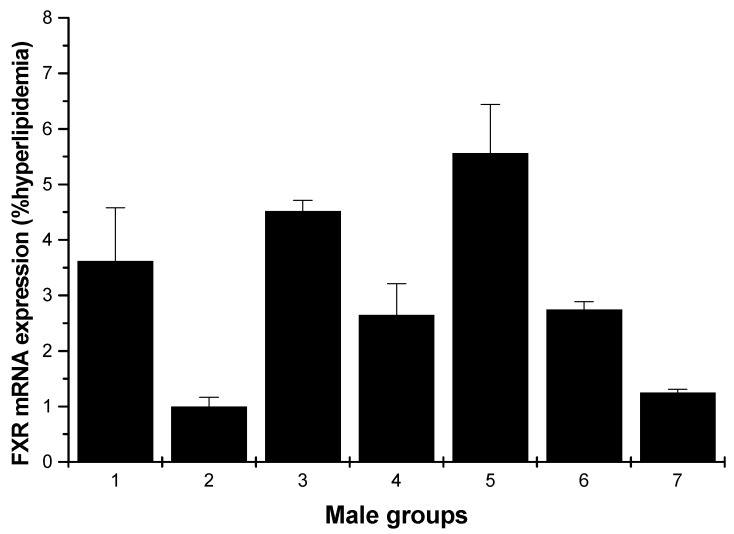
FXR mRNA expression in the livers of male rats (hyperlipidemia group as stand one). 1: normal control group; 2: hyperlipidemia group; 3: ulvan group (250 mg/kg); 4: HU low-dose group (125 mg/kg); 5: HU middle dose group (250 mg/kg); 6: HU high-dose group (500 mg/kg); 7: positive group (cholestyramine, 500 mg/kg).

**Figure 6:**
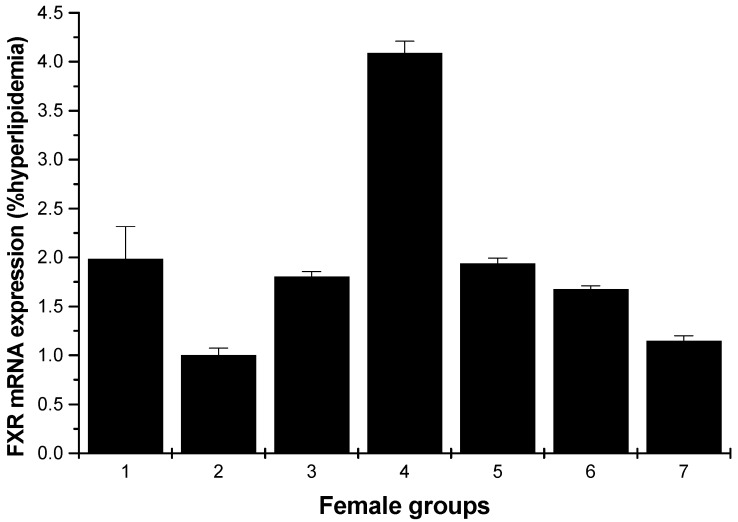
FXR mRNA expression in the livers of female rats (hyperlipidemia group as stand one). 1: normal control group; 2: hyperlipidemia group; 3: ulvan group (250 mg/kg); 4: HU low-dose group (125 mg/kg); 5: HU middle dose group (250 mg/kg); 6: HU high-dose group (500 mg/kg); 7: positive group (cholestyramine, 500 mg/kg).

The effects of various samples on FXR mRNA expression are shown in Figure 5 and Figure 6. In Figure 5, for the male rats, compared with the hyperlipidemia group, all groups increased the FXR mRNA expression, especially the middle dose (250 mg/kg) HU group, which had the most pronounced effect with respect to this increasing (*p* < 0.01).

**Table 1:** Effects of U, HU and cholestyramine on serum lipid profiles in rats supplemented with a cholesterol-rich diet.

Group	Dose (mg/kg)	TC (mmol/L)	TG (mmol/L)	LDL-C (mmol/L)	HDL-C (mmol/L)
Male					
Normal control	-	1.69 ± 0.15	0.87 ± 0.13	0.49 ± 0.03	1.04 ± 0.04
Hyperlipidemia	-	2.21 ± 0.25 ^Δ^	1.23 ± 0.11 ^Δ^	0.70 ± 0.04 ^Δ^	0.82 ± 0.13 ^Δ^
U	250	1.34 ± 0.35 *	0.85 ± 0.05	0.41 ± 0.01 *	0.81 ± 0.05
HU (low dose)	125	1.34 ± 0.06 *	0.79 ± 0.02	0.41 ± 0.02 *	0.88 ± 0.02
HU(middle dose)	250	1.30 ± 0.10 **	0.72 ± 0.14 *	0.43 ± 0.05	0.93 ± 0.13
HU (high dose)	500	1.39 ± 0.18	0.78 ± 0.06	0.43 ± 0.04	0.99 ± 0.01 *
Positive control	500	1.77 ± 0.18	0.91 ± 0.22	0.50 ± 0.06	1.11 ± 0.07 **
Female					
Normal control	-	1.89 ± 0.13	0.72 ± 0.03	0.41 ± 0.03	1.20 ± 0.09
Hyperlipidemia	-	2.25 ± 0.47 ^Δ^	1.21 ± 0.09 ^Δ^	0.60 ± 0.05 ^Δ^	0.84 ± 0.25 ^Δ^
U	250	1.63 ± 0.30 *	0.76 ± 0.08	0.38 ± 0.06	0.98 ± 0.14
HU (low dose)	125	1.26 ± 0.04 **^,#^	0.65 ± 0.04 *	0.30 ± 0.01 **	1.05 ± 0.07 *
HU(middle dose)	250	1.26 ± 0.04 **^,#^	0.67 ± 0.05 *	0.32 ± 0.01	0.91 ± 0.07
HU (high dose)	500	1.42 ± 0.11	0.67 ± 0.07 *	0.31 ± 0.05 *	0.91 ± 0.09
Positive control	500	1.75 ± 0.17	0.81 ± 0.16	0.38 ± 0.02	1.15 ± 0.12 **

^Δ ^*p* < 0.05: compared with the normal control group; * *p* < 0.05: compared with the hyperlipidemia control group; ** *p* < 0.01: compared with the hyperlipidemia control group; ^#^ *p* < 0.05: compared with the ulvan group.

#### 2.1.4. LXR mRNA Expression

**Figure 7:**
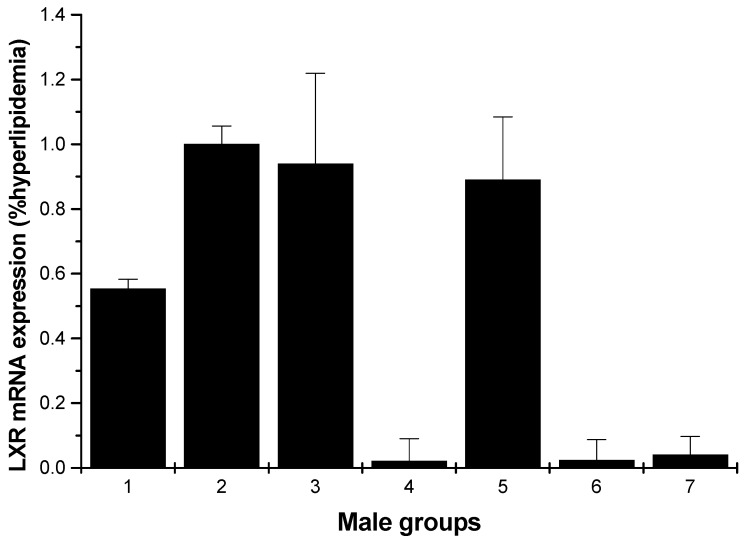
LXR mRNA expression in the livers of male rats (hyperlipidemia group as stand one). 1: normal control group; 2: hyperlipidemia group; 3: ulvan group (250 mg/kg); 4: HU low-dose group (125 mg/kg); 5: HU middle dose group (250 mg/kg); 6: HU high-dose group (500 mg/kg); 7: positive group (cholestyramine, 500 mg/kg).

**Figure 8:**
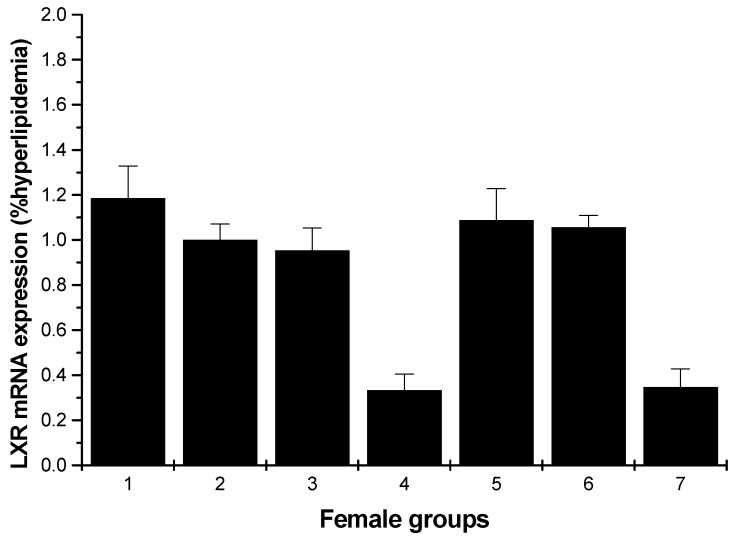
LXR mRNA expression in the livers of female rats (hyperlipidemia group as stand one). 1: normal control group; 2: hyperlipidemia group; 3: ulvan group (250 mg/kg); 4: HU low-dose group (125 mg/kg); 5: HU middle dose group (250 mg/kg); 6: HU high-dose group (500 mg/kg); 7: positive group (cholestyramine, 500 mg/kg).

Figure 7 shows a significant inhibition of the mRNA levels of LXR compared to the hyperlipidemia group for the male rats (low- and high-dosage HU groups, *p* < 0.01). For the female rats, except for the normal control, middle and high-dose HU groups, others showed inhibition on the LXR mRNA levels; moreover, the enhancements of the normal control, middle and high-dose HU groups were weak (Figure 8).

#### 2.1.5. PPARγ mRNA Expression

**Figure 9:**
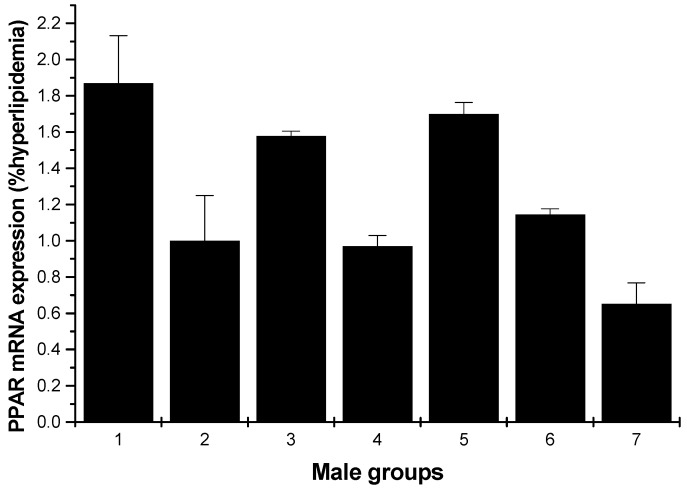
PPARγ mRNA expression in the livers of male rats (hyperlipidemia group as stand one). 1: normal control group; 2: hyperlipidemia group; 3: ulvan group (250 mg/kg); 4: HU low-dose group (125 mg/kg); 5: HU middle dose group (250 mg/kg); 6: HU high-dose group (500 mg/kg); 7: positive group (cholestyramine, 500 mg/kg).

**Figure 10:**
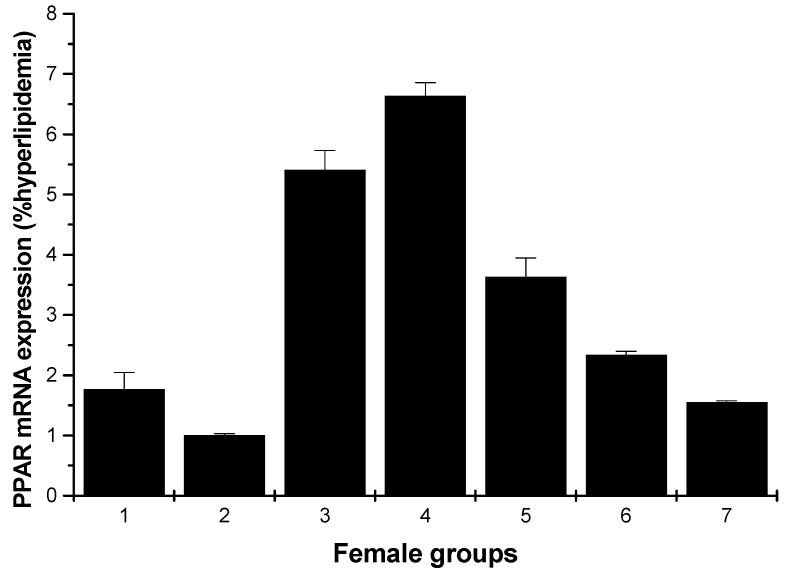
PPARγ mRNA expression in the livers of female rats (hyperlipidemia group as stand one). 1: normal control group; 2: hyperlipidemia group; 3: ulvan group (250 mg/kg); 4: HU low-dose group (125 mg/kg); 5: HU middle dose group (250 mg/kg); 6: HU high-dose group (500 mg/kg); 7: positive group (cholestyramine, 500 mg/kg).

Figure 9 and Figure 10 depict the effect of all samples on PPARγ mRNA expression. For the male rats, all groups could enhance the mRNA expression on PPARγ, except for the low-dose HU and cholestyramine groups compared to the hyperlipidemia group (Figure 9). However, for the female rats, all groups showed increased mRNA expression on PPARγ; furthermore, the effect was in a concentration-dependent manner (Figure 10).

### 2.2. Discussion

Hyperlipidemia is one key factor causing atherosclerosis fatty liver and cardio-cerebrovascular diseases; hence, consumption of polysaccharides with antihyperlipidemic activity could reduce the risk of associated diseases [16]. Fan *et al.* reported that okra polysaccharide decreased serum total cholesterol levels in high-fat diet-fed C57BL/6 mice [17]. The extracellular polysaccharide LEP-1b produced by Lachnum YM281 had a strong lipid lowering and liver protecting effect on mice with hyperlipidemic fatty liver [18]. 

FXR is a member of the nuclear hormone receptor super-family. High expression of FXR is limited to the liver, intestine, kidney and adrenal gland [19,20,21]. It has been shown to be important in controlling numerous metabolic pathways; these include roles in maintaining bile acid, glucose and lipid homeostasis, in preventing intestinal bacterial infection and gallstone formation and in modulating liver regeneration and tumorigenesis [22]. In the current study, these results described above indicated that HU can effectively reduce the lipid levels; at the same time, the results proved that the HU groups demonstrated the activity of upregulating the expression of FXR. Especially, the middle dose HU group of male rats and the low-dose HU group of female rats showed the most pronounced increasing ability (Figure 5 and Figure 6). That is to say, HU had a strong antihyperlipidemic activity that may be through the upregulating of the expressions of the mRNA FXR. In addition, activation of FXR lowers plasma triglyceride levels [23,24] by a mechanism that involves the repression of hepatic SREBP-1c expression [25,26]. Qiu *et al.* reported that LEP-1b can effectively reduce the hepatic lipid level, and the reason may be that LEP-1b was due to the upregulating of the expressions of ileum FXR [18].

An early study reported that LXRs exist as two isoforms, LXRα and LXRβ, and they differ mainly in their tissue distribution. LXRα is expressed extensively in the liver and fat and at lower levels in the intestine, macrophages, lung, adrenal glands and kidney. In contrast, LXRβ is expressed ubiquitously [27]. To determine the mechanism of HU for antihyperlipidemic activity, we assayed the gene expression levels of nuclear receptor transcription factors in the rats’ liver. LXRs are important nuclear receptor transcription factors, which can regulate lipid and glucose homeostasis [28]. As shown in Figure 7, for male rats, all samples proved an inhibition of the mRNA levels of LXRα compared to the hyperlipidemia group, especially the low-dose and high-dose groups. Figure 8 indicates weak enhancing effect for some samples. Fan *et al.* reported that okra polysaccharide may have therapeutic effects on metabolic diseases via the inhibition of LXR [17]. In light of this consistent finding, it would be of interest to find out if HU exerts its antihyperlipidemic activity by downregulating LXRs.

PPARs occur as three isoforms: PPARα, PPARγ and PPARδ. PPARγ is highly distributed in skeletal muscle, liver and adipose tissue. PPARγ agonists, such as thiazolidinediones or glitazones, have been used for treating insulin resistance and hyperglycemia [4]. To further evaluate the possible mechanism, the effects of HU on lipid metabolic gene PPARγ expression of hyperlipidemic rats were analyzed using RT-PCR. In the present study, ulvan and the different dose of HU groups all showed an upregulation effect on mRNA expression of PPARγ, in female rats; the low-dose HU group proved to have the strongest increasing effect. Yu *et al.* reported that the polysaccharide extracted from *Rosae Laevigatae Fructus* RLP-1 was responsible for the hypolipidemic effect and could improve lipid profiles through upregulation of PPARγ expression [29]. It has been reported that PPARγ agonists upregulate human macrophage LPL expression [30]; whether HU could increase PPARγ mRNA expression thorough upregulation of LPL expression needs to be studied further.

Abundant data suggested that the activity of polysaccharide depends on several structural parameters, such as the molecular weight, the degree of sulfation (DS), the sulfation position, type of sugar and glycosidic branching [9,13,31,32]. In the present study, the sulfate content of HU was higher than that of U, 32.8% and 19.5%, respectively. The results indicated that HU had stronger hyperlipidemic activity than U; at the same time, upregulation of the mRNA expression of FXR and PPARγ by HU was stronger than that of U. It may be concluded that the high sulfate content of polysaccharide could improve the antihyperlipidemic activity through upregulation of the expression of FXR and PPARγ.

The reason may be that HU has the capacity to inhibit the entero-hepatic cycle of bile acids and to enhance the metabolism and decomposition of cholesterols through upregulation of the expression of ileum FXR and hepatic CYP7A1 genes and downregulation of the expression of 1-BABP and hepatic FXR. Another reason may be that the transcriptional upregulation of PPARγ by HU leads to the decrease of the fat synthesis rate and the increase of the fat hydrolysis rate, thus accelerating the metabolism and decomposition of triglyceride [33,34,35]. However, the specific mechanism of the lipid lowering of HU needs further investigation.

Thus, to our knowledge, the present study provides the first evidence that HU could affect FXR, LXR and PPARγ signaling. The exact mechanism of HU on the target gene signaling needs further investigation. On the other hand, the effects of HU on the protein expression of the target genes need further study.

## 3. Experimental Section

### 3.1. Materials and Reagents

*U. pertusa* was collected in the coast of Qingdao, China, in October 2013. The fresh algae were promptly washed, sun dried and kept in plastic bags at room temperature for use. The assay kits for serum cholesterol (TC), triacylglycerols (TG), high-density lipoprotein cholesterol (HDL-C) and low-density lipoprotein cholesterol (LDL-C) were purchased from Shanghai Rongsheng (China). Other reagents, unless stated otherwise, were analytical grade and purchased from Weifang Runze Co. Ltd. (Weifang, China).

### 3.2. Preparation of Natural Polysaccharide Ulvan

Ulvan was extracted from *U. pertusa*, collected in the coast of Qingdao, China, as described previously [9]. Generally, dry algae were cut roughly and autoclaved in water at 125 °C for 4 h. The hot aqueous solution was separated, concentrated, dialyzed, precipitated and then dried to give polysaccharide, named U (yield 22.5%).

### 3.3. Preparation of High Sulfate Content Ulvan HU

The ulvan derivative HU was prepared as described previously [9]. Generally, first, the sulfation agent, SO_3_–DMF, was prepared. Then, dry ulvan was added to formamide (FA), and the mixture was stirred at 60 °C for 30 min. SO_3_–DMF reagent was added. After 4 h, the mixture was cooled, neutralized with NaOH solution, precipitated, dissolved in distilled water, dialyzed, concentrated and lyophilized to give HU (yield 40.6%).

### 3.4. Chemical Analysis

Sulfate content was determined by using the traditional method of barium chloride-gelatin [36]. The sulfate contents of U and HU were 19.5% and 32.8%, respectively. At the same time, the HU was prepared successfully according the FTIR and ^13^C-NMR spectrums. In the ^13^C-NMR spectrum, the results indicated that hydroxyl groups of C-2 (R, R′ and I) and C-3 (I) were partly sulfated [9].

### 3.5. Animals and Experimental Design

#### 3.5.1. Animals

The studies were conducted at the Laboratory of Pharmacology and approved by Ethics Committee of Weifang Medical University. Eighty-four Wistar rats (male/female, 180–220 g) were provided by the Animal Lab Center of Shandong University (animal license: SCXK (Lu) 20090001) (China). The animal room was maintained at a temperature of 25 ± 2 °C, a relative humidity of 50% ± 15%, a light/dark cycle of 12 h (fluorescent light) and ≥10 air changes per hour. The animals were allowed free access to standard laboratory pellet diet and water during the experiments.

Rats were fed with basic diet for 7 days in the experimental environment before the experiments were conducted. Once they had adapted to the environment, a total of 12 rats were selected randomly as the normal control group fed with a basic diet, whereas the others were fed with a high-cholesterol diet (2.0% cholesterol, 8.0% lard, 0.3% sodium cholic acid and 89.7% commercial chow) and were randomly divided into 6 groups: the hyperlipidemia model control group, the ulvan group (250 mg/kg body weight), the HU low-dosage group (125 mg/kg body weight), the HU middle-dosage group (250 mg/kg body weight), HU the high-dosage group (500 mg/kg body weight) and the positive control group (cholestyramine, 500 mg/kg body weight). Based on the preliminary activity, toxicity experiments and the solubility of HU, the dosages were confirmed at 125, 250 and 500 mg/kg body weight. The normal control group and hyperlipidemia model group were orally administered with the same volume of water, whereas the other groups were treated with corresponding drugs once daily for 30 days. Rats had free access to water and food *ad libitum*. During the experiment, body weights of the rats were weighed every 6 days, and the food intakes were measured every day. 

#### 3.5.2. Determination of TC, TG, LDL-C and HDL-C Levels

At the end of the experimental period (30 days), food was withheld from the rats for at least 12 h. They were weighed and had blood samples collected from the eyeballs, then serum was isolated by centrifugation at 3000 rpm for 10 min. The levels of TC, TG, HDL-C and LDL-C were determined by the described kits’ methods.

#### 3.5.3. RT-PCR Analysis

At the end of the experimental period (30 days), food was withheld from the rats for at least 12 h. They were weighed, and the livers were removed, rinsed with physiological saline and immediately stored at −70 °C.

Total RNA was obtained from rat liver tissue using TRIzol Reagent (Invitrogen, Carlsbad, CA, USA) following the instructions of the manufacturer. The cDNA was synthesized from 2 mg of total RNA with M-MuLV reverse transcriptase and random 18 oligo dT according to the supplier’s instructions (Takara, Otsu, Japan). Then, the cDNA products were stored at −20 °C until use. Next, triplicate cDNA samples were assessed for target mRNA levels by real-time quantitative reverse transcriptase polymerase chain reaction (qRT-PCR) with SsoFast EvaGreen Supermix (Bio-Rad, Hercules, CA, USA) on a Bio-Rad IQ5 real-time PCR system. The PCR conditions were 30 cycles of 95 °C for 30 s, 60 °C for 30 s and 72 °C for 30 s. Target cDNA amplification was normalized to β-actin expression, and the relative levels of target mRNA levels were calculated. The primer sequences were as follows in Table 2.

**Table 2:** The primers of gene and β actin.

Gene	Forward Primers	Reverse Primers
FXR	5′-AGCCCGAGAACCCTCAGCATT-3′	5′-TCATTCACCCTCCAAGACATCAGC-3′
LXR	5′-GAACGAGCTATGCAGTGTATGTGGG-3′	5′-GCTCCTCTTCTTGACGCTTCAGTTT-3′
PPARγ	5′-CAAGGAGGCAGAGGTCCGATTC-3′	5′-CTTGGGTTCCATGATGTCGCAG-3′
β actin	5′-GAACCCTAAGGCCAACCGTGAA-3′	5′-CGACCAGAGGCATACAGGGACA-3′

### 3.6. Statistical Analysis 

The data were presented as the means ± SD and evaluated by one-way ANOVA followed by Student’s *t*-test to detect inter-group differences. Differences were considered to be statistically significant if *p* < 0.05.

## Abbreviations

U: ulvan
HU: high sulfate content ulvan
SO_3_–DMF: sulfur trioxide/*N*,*N*-dimethylformamide
TC: total cholesterol
TG: triglyceride
HDL-C: high-density lipoprotein cholesterol
LDL-C: low-density lipoprotein cholesterol
FXR: farnesoid X receptor
LXR: liver X receptor
PPAR: peroxisome proliferator-activated receptor
